# Association between *Plasmodium* Infection and Nitric Oxide Levels: A Systematic Review and Meta-Analysis

**DOI:** 10.3390/antiox12101868

**Published:** 2023-10-16

**Authors:** Kwuntida Uthaisar Kotepui, Aongart Mahittikorn, Polrat Wilairatana, Frederick Ramirez Masangkay, Manas Kotepui

**Affiliations:** 1 Medical Technology, School of Allied Health Sciences, Walailak University, Tha Sala, Nakhon Si Thammarat 80160, Thailand; kwuntida.ut@wu.ac.th; 2 Department of Protozoology, Faculty of Tropical Medicine, Mahidol University, Bangkok 10400, Thailand; aongart.mah@mahidol.ac.th; 3 Department of Clinical Tropical Medicine, Faculty of Tropical Medicine, Mahidol University, Bangkok 10400, Thailand; 4 Department of Medical Technology, Faculty of Pharmacy, University of Santo Tomas, Manila 1008, Philippines

**Keywords:** malaria, systematic review, meta-analysis, nitric oxide, *Plasmodium*

## Abstract

Nitric oxide (NO) has been implicated in the pathology of malaria. This systematic review and meta-analysis describe the association between NO levels and malaria. Embase, Ovid, PubMed, Scopus, and Google Scholar were searched to identify studies evaluating NO levels in malaria patients and uninfected controls. Meta-regression and subgroup analyses were conducted to discern differences in NO levels between the groups. Of the 4517 records identified, 21 studies were included in the systematic review and meta-analysis. The findings illustrated significant disparities in NO levels based on geographic location and study time frames. Despite the fluctuations, such as higher NO levels in adults compared to children, no significant differences in mean NO levels between patients and uninfected controls (*p* = 0.25, Hedge’s g: 0.35, 95% confidence interval (CI): −0.25–0.96, *I*^2^: 97.39%) or between severe and non-severe malaria cases (*p* = 0.09, Hedge’s g: 0.71, 95% CI: −0.11–1.54, *I*^2^: 96.07%) were detected. The systematic review and meta-analysis highlighted inconsistencies in NO levels in malaria patients. Given the high heterogeneity of the results, further studies using standardized metrics for NO measurements and focusing on biochemical pathways dictating NO responses in malaria are imperative to understand the association between NO and malaria.

## 1. Introduction

Malaria is one of the world’s most critical infectious diseases, posing considerable public health challenges, especially in tropical and subtropical regions where malaria is endemic [1]. Malaria, which is primarily transmitted through the bite of infected female *Anopheles* mosquitoes, is caused by *Plasmodium* parasites. *P. falciparum* and *P. vivax* are the most prominent species causing malaria infections globally [1,2,3]. Despite concerted global health efforts to curb the disease, malaria continues to exact a heavy toll, with hundreds of thousands succumbing to the illness annually, including children under the age of five [1,4]. In addition to developing vaccines and potent antimalarial drugs, understanding the underlying biological mechanisms and responses to the infection is a focal point of research into malaria.

Nitric oxide (NO) is a soluble gas that plays a crucial role in numerous physiological and pathological processes in humans [5,6]. NO regulates vasodilation, neurotransmission, and immune responses in many physiological systems, including the cardiovascular, nervous, and immune systems [7,8]. NO is produced endogenously from L-arginine by nitric oxide synthases (NOSs). The functions of NO range from the regulation of blood pressure to the modulation of immune responses [8,9]. In addition, NO is a potent antimicrobial agent within the host’s innate immune system [10]. Upon infection, inducible NOS is upregulated in immune cells, leading to increased production of NO [11]. The high concentrations of NO directly inhibit replication or kill various pathogens, including bacteria, viruses, fungi, and parasites [12,13,14,15].

NO produced by host immune cells plays a dual role in malaria. Elevated NO levels restrict the growth of *Plasmodium* parasites and prevent propagation within the host [16]. NO elicits antiparasitic effects by disrupting parasitic biochemical pathways and inducing oxidative stress that hampers survival [17]. Furthermore, NO modulates the host immune response by regulating proinflammatory cytokine production and enhancing the phagocytic activity of immune cells against the malaria parasite [18,19]. However, excessive NO production can be detrimental. Excessive NO production induces oxidative stress in the host, leading to tissue damage and exacerbating disease symptoms [18,20]. This delicate balance between the beneficial and detrimental effects of NO highlights the importance of tightly regulating NO production during infection.

Exploring the association between nitric oxide levels and malaria presents a nuanced yet vital avenue for understanding the pathology of malaria. Over the past decades, the complex relationship between NO and the pathology of malaria has been highlighted in many studies. On the one hand, high NO levels produced by the host immune system are associated with controlling parasite density and protecting against severe disease manifestations [16,21,22]. Conversely, excessively elevated NO levels have been linked to increased parasitemia [17,23], leading to severe malaria complications. Therefore, unraveling the intricate relationship between NO levels and the severity of malaria is a crucial part of contemporary research that may lead to novel insights and therapeutic strategies for treating malaria. This systematic review and meta-analysis aimed to analyze existing research and gain a more cohesive understanding of the role of NO in malaria patients, paving the way for informed future investigations.

## 2. Materials and Methods

### 2.1. Protocol and Registration

The research framework and strategy adhered to the PRISMA (Preferred Reporting Items for Systematic Reviews and Meta-Analyses) guidelines to ensure a high standard of reporting [24]. Furthermore, the protocol for this review was registered with PROSPERO (registration number: CRD42023457918).

### 2.2. Review Question

The framework of this systematic review was constructed following the Population, Exposure, Comparator, Outcome (PECO) approach [25], encompassing the following components: P was the participants in the studies, E was the *Plasmodium* infection or severe disease, C was the uninfected controls or less severity, and O was the NO levels.

### 2.3. Literature Search 

The literature review was comprehensively performed to identify studies investigating NO levels in malaria patients compared to uninfected controls and across different malaria severity levels. Embase, Ovid, PubMed, Scopus, and Google Scholar were searched using the following specific keywords and phrases: “Nitric oxide” OR “Nitrogen Monoxide” OR “Endothelium-Derived Nitric Oxide” OR “Endogenous Nitrate Vasodilator” OR “Mononitrogen Monoxide” AND “malaria” OR “Plasmodium” OR “Plasmodium Infection” OR “Remittent Fever” OR “Marsh Fever” OR “Paludism” (detailed in Table S1). Additional searches were carried out in Google Scholar and the reference lists of the included studies. The search period extended from the inception of the databases to 25 August 2023. There were no restrictions based on language or publication date.

### 2.4. Study Selection

Studies were eligible for inclusion in the systematic review and meta-analysis if they reported NO levels in patients with malaria and uninfected individuals, or patients experiencing varying severity levels of malaria. All study designs were considered, including clinical trials that presented baseline data on NO levels in malaria patients. Studies were excluded if they were in vivo studies, reviews, case reports, case series, systematic reviews, letters to the editor, in vitro studies, mosquito studies, lacked NO information, contained NO data that could not be extracted, or were unavailable in full-text format. Two authors independently selected the studies, and disagreements were resolved through discussion to reach a consensus.

### 2.5. Data Extraction

The data were meticulously extracted from the selected studies. The following data were retrieved: publication years, study design, geographic location of the study, age of participants, *Plasmodium* species, diagnostic methods for malaria determination, NO measurement methods, and the types of blood samples used. Two authors independently extracted the data, and disagreements were resolved through discussion to reach a consensus.

### 2.6. Risk of Bias

The Joanna Briggs Institute (JBI) critical appraisal tools for cross-sectional, cohort, and case–control studies were employed to assess each study across specific criteria [26]. The checklist for cross-sectional studies includes eight criteria focused on sample inclusion, description of subjects and setting, measurement reliability, confounder management, and statistical analysis. The checklist for case–control studies comprise ten criteria, including group comparability, case-control matching, and standard exposure and outcome assessments. The cohort studies checklist consists of eleven criteria, including group similarity and recruitment, exposure, outcome measurement validity, and statistical analysis appropriateness. Each checklist ensures a rigorous and standardized evaluation of the respective studies.

### 2.7. Thematic Analysis

A qualitative synthesis of the collected data was performed through a thematic analysis approach [27]. The data were grouped into themes based on the comparison of NO levels between patients with malaria and uninfected individuals, and the variance in NO levels according to the severity of malaria infection. The synthesis was based on a detailed inspection of individual study findings to construct thematic discussions. The results of the thematic synthesis were tabulated and narratively summarized.

### 2.8. Meta-Analysis

Studies were included in the meta-analysis if they reported NO levels as means or medians accompanied by standard deviations or ranges. The outcome of interest was the difference in NO levels between the two groups. Differences in NO levels between patients with severe and non-severe forms of malaria were also analyzed. Hedge’s g was used to determine the effect size, and the results were presented with a 95% confidence interval (CI). Heterogeneity between studies was assessed using the *I*^2^ statistic; 0–40% indicated low heterogeneity, 30–60% indicated moderate heterogeneity, 50–90% indicated substantial heterogeneity, and 75–100% indicated considerable heterogeneity [28]. High heterogeneity was observed between studies, and meta-regression and subgroup analyses were conducted to explore potential sources of heterogeneity. The meta-regression analyses included publication years, study design, continent, age group, *Plasmodium* species, diagnostic methods for malaria, methods for NO measurement, and types of blood samples. The influence of these factors on the pooled effect estimate was assessed. Based on these variables, subgroup analyses were conducted. The results of the meta-analysis are presented as forest plots. All statistical analyses were performed utilizing Stata v17.0 software (StataCorp., College Station, TX, USA), with *p*-values less than 0.05 deemed statistically significant.

## 3. Results

### 3.1. Search Results

**Figure 1:**
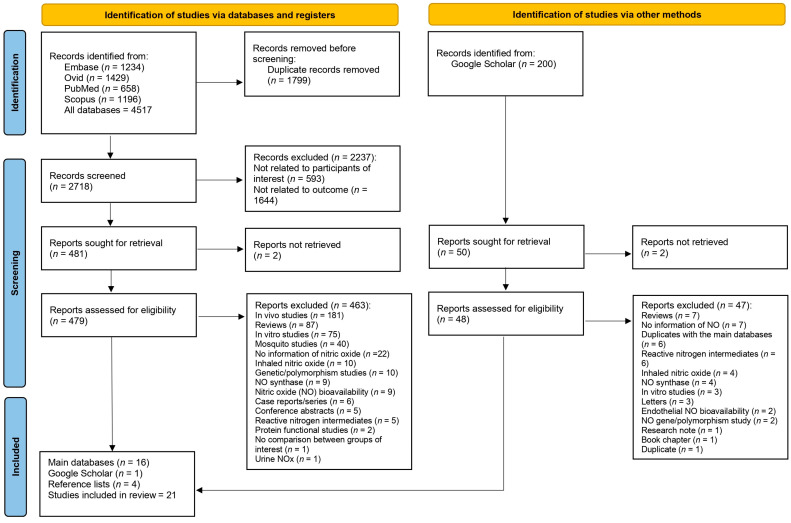
The study selection process.

A total of 4517 records were initially identified from the following databases: Embase (*n* = 1234), Ovid (*n* = 1429), PubMed (*n* = 658), and Scopus (*n* = 1196). Duplicate records (*n* = 1799) were removed, leaving 2718 records for screening. Records (*n* = 2237) were excluded for the following reasons: 593 records were not related to the participants of interest, and 1644 records were not related to the outcome. Of the remaining 481 reports, 2 could not be retrieved. The remaining 479 reports were assessed for eligibility. Studies were excluded (*n* = 463) for the following reasons: 181 were in vivo studies, 87 were reviews, 75 were in vitro studies, 40 were mosquito studies, and the rest were excluded for other reasons such as no information on NO (*n* = 22), inhaled NO (*n* = 10), and genetic/polymorphism studies (*n* = 10). Finally, 21 studies were included in the review [16,23,29,30,31,32,33,34,35,36,37,38,39,40,41,42,43,44,45,46,47]. These studies were sourced from the main databases (*n* = 16), Google Scholar (*n* = 1), and reference lists (*n* = 4) (Figure 1).

### 3.2. Characteristics of Included Studies

Table 1 summarizes the characteristics of the 21 studies included in the analysis. The range of publication years was diverse, with 33.33% published before 2000, 19.05% between 2000 and 2009, 38.10% between 2010 and 2019, and 9.52% between 2020 and 2023. The studies employed various designs, including case–control (33.33%), cross-sectional (42.86%), and cohort studies (23.81%). Geographically, most studies were conducted in Africa (66.67%), particularly in Nigeria and Cameroon, followed by Asia (14.29%), South America (9.52%), and Europe (9.52%). *P. falciparum* was the most common *Plasmodium* species studied (76.19%). The participant ages were evenly split between children and adults (42.86% each); only 9.52% of the studies included both children and adults. Most studies focused on symptomatic malaria (66.67%). The microscopic method was used for *Plasmodium* detection in most studies (52.38%). The Griess assay was the most frequently used method for NO measurement (57.14%), and plasma was the predominant choice for blood sampling for NO measurement (57.14%).

### 3.3. Risk of Bias

Nine studies were evaluated using the JBI critical appraisal checklist for analytical cross-sectional studies [16,23,32,35,39,41,42,44,45]. All of the studies met the JBI criteria, except the Megnekou et al. 2015 study [41]; the identification and strategies for dealing with confounding factors were unclear in this study. Seven studies were assessed using the JBI critical appraisal checklist for case–control studies [29,30,33,34,43,46,47]. Most studies met all of the criteria. However, the identification of confounding factors was unclear in Agbenyega et al. [29] and Nsonwu-Anyanwu et al. [43], and confounding factors were not identified, strategies to deal with confounding factors were not stated, and the appropriateness of statistical analysis was unclear in Arun Kumar C and Das UN [30], De Sousa et al. [33], and Torre et al. [46]. Five studies were assessed using the JBI critical appraisal checklist for cohort studies [31,36,37,38,40]. Gyan et al. [36] met all of the criteria. The identification of confounding factors was unclear, no strategies were stated to deal with confounding factors, and follow-up was inadequate and incomplete in Awalu et al. [31] and Inocent et al. [37]. The confounding factors were unclear and follow-up was incomplete in Kremsner et al. [38] and Lima-Junior et al. [40] (Table S2).

### 3.4. Qualitative Synthesis by the Thematic Analysis

The NO levels were compared between patients with malaria and uninfected controls (Table 2). NO levels were significantly higher in patients with malaria compared to uninfected controls in several studies, including Awalu et al. in Nigeria [31], De Sousa et al. in Portugal [33], Ojongnkpot et al. in Cameroon [44], Lima-Junior et al. in Brazil [40], and Kumar et al. in India [39]. In the study by Ojongnkpot et al., NO levels were higher in patients with symptomatic malaria compared to asymptomatic malaria [44]. Cramer et al. [32] and Gyan et al. [36] specifically noted elevated NO levels in severe forms of the disease, such as severe malarial anemia. In contrast, lower NO levels were reported in malaria patients compared to controls in Anstey et al. from Tanzania [16] and Arun Kumar C and Das UN from India [30], particularly in patients with uncomplicated malaria and cerebral malaria. Nsonwu-Anyanwu et al. from Nigeria also reported significantly lower NO levels in malaria patients compared to uninfected individuals [43]. A substantial number of studies, including Agbenyega et al. [29], Inocent et al. [37], Dongho Dongmo et al. [35], Megnekou et al. [41], Noone et al. [42], Onyeneke et al. [47], Sánchez-Arcila et al. [45], and Torre et al. [46], found no significant differences in NO levels between patients with malaria and uninfected controls. 

The NO levels were compared between patients with different malaria severity levels. No significant differences in the NO levels between severe and non-severe malaria cases, or between survivors and fatal cases were detected in several studies. For example, no differences in NO concentrations between severe and non-severe cases of *P. falciparum* malaria were detected in a study by Agbenyega et al. conducted in children in Ghana [29]. Similarly, Inocent et al. in Cameroon detected no significant differences in the NO levels between severe malaria and non-severe cases among children aged 0–15 years [37]. No significant differences in the NO levels between survivors and fatal cases of *P. falciparum* malaria were detected in a study by Dondorp et al. in Thailand [34]. In a study in Cameroon, Dongho Dongmo et al. observed no differences in NO concentrations among cerebral malaria, malaria anemia, and uncomplicated malaria cases in children aged 15 years or younger [35]. In contrast to these studies, Anstey et al. reported that NO was significantly higher in asymptomatic individuals with malaria compared with asymptomatic individuals without malaria based on thick film microscopy [23].

### 3.5. NO Levels in Patients with Malaria and Uninfected Controls

**Figure 2:**
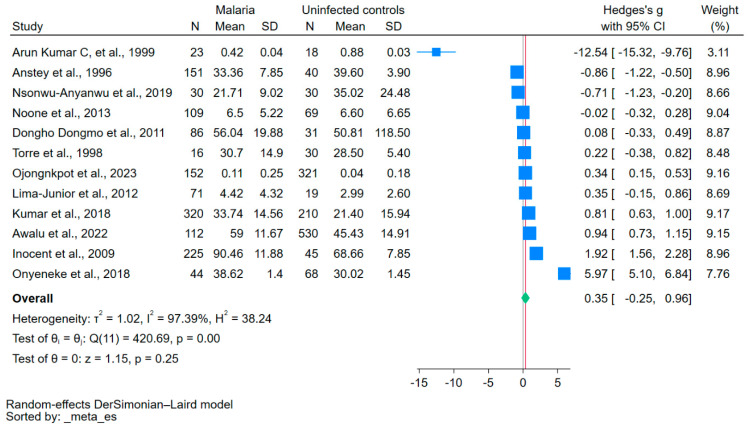
Forest plot of meta-analysis comparing NO levels between patients with malaria and uninfected controls. The mean NO levels were similar between the two groups (*p* = 0.25). The blue boxes indicate effect estimates for individual studies; the green diamond indicates the pooled effect estimate. N, number of participants in each group; SD, standard deviation; CI, confidence interval. References [16,30,31,35,37,39,40,42,43,44,46,47].

The NO levels in patients with malaria and uninfected controls were compared using a meta-analysis of 12 studies that reported NO levels as means or medians with standard deviations or ranges [16,30,31,35,37,39,40,42,43,44,46,47]. The mean NO levels were similar between the two groups (*p* = 0.25, Hedge’s g: 0.35, 95% CI: −0.25–0.96, *I*^2^: 97.39%, Figure 2). 

A high level of heterogeneity (*I*^2^: 97.39%) was observed in the studies included in the meta-analysis. Consequently, meta-regression and subgroup analyses were conducted to explore the sources of heterogeneity. The meta-regression analysis incorporated factors such as publication years, study design, continent, age group, *Plasmodium* species, diagnostic methods for malaria, methods for NO measurement, and types of blood samples. Publication years, continent, age group, methods for NO measurement, and types of blood samples were identified as factors that significantly influenced the pooled effect estimate (*p* < 0.05, Table S4).

The subgroup analyses are shown in Table 3. Differences in the NO levels in patients with malaria and uninfected controls varied considerably, depending on several factors. Studies published between 2020 and 2023 showed a moderate but significant increase in NO levels in patients with malaria compared with uninfected controls (*p* = 0.03, Hedges’ g = 0.64). In contrast, studies published before 2000 showed that NO levels significantly decreased in patients with malaria compared with uninfected controls (*p* = 0.01, Hedges’ g = −3.52). In terms of study design, case–control studies did not demonstrate significant differences in NO levels (*p* = 0.44), whereas cohort studies presented a high Hedges’ g value of 1.09, although the number of studies was limited. Geographically, studies from Africa demonstrated a significant increase in NO levels (*p* = 0.02), while studies from Asia did not demonstrate any differences (*p* = 0.39). A significant increase in NO levels was noted in adults (*p* = 0.01), but not in children (*p* = 0.45). A significant increase in NO levels was detected in studies involving multiple *Plasmodium* species, such as *P. falciparum* and *P. vivax* (*p* < 0.01). No significant differences in NO levels were detected between symptomatic versus asymptomatic or severe versus non-severe cases. Specifically, symptom severity did not yield notable differences (*p* = 0.65 for severe and uncomplicated malaria; *p* = 0.04 for non-severe malaria). A significant increase in NO levels was noted in studies that employed the colorimetric method for detecting NO (though the specific assay was not stated) (*p* < 0.01). Studies using serum samples demonstrated a significant increase in NO levels (*p* < 0.01), while those using plasma did not show a significant difference (*p* = 0.10).

### 3.6. NO Levels in Patients with Severe and Non-Severe Malaria 

**Figure 3:**
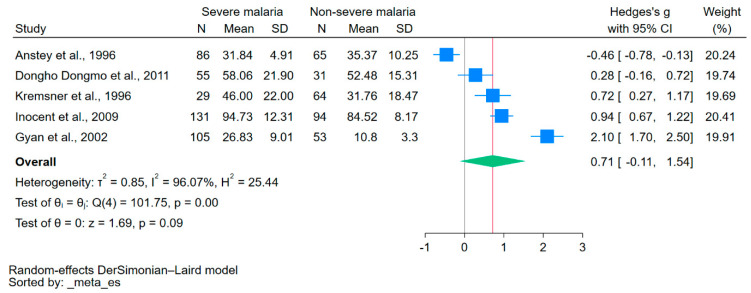
Forest plot of meta-analysis comparing NO levels between patients with severe and nonsevere malaria. Mean NO levels were similar between the two groups (*p* = 0.09). The blue boxes indicate the effect estimates for individual studies, and the green diamond indicates the pooled effect estimate. N, number of participants in each group; SD, standard deviation; CI, confidence interval. References [16,35,36,37,38].

In the meta-analysis, the NO levels in patients with severe and non-severe malaria were compared. The analysis incorporated data from five studies that reported NO levels as means or medians with standard deviations or ranges [16,35,36,37,38]. The mean NO levels were similar between the two groups (*p* = 0.09, Hedge’s g: 0.71, 95% CI: −0.11–1.54, *I*^2^: 96.07%, Figure 3). Meta-regression and subgroup analyses were not conducted due to the low number of studies in the meta-analysis.

### 3.7. Publication Bias 

**Figure 4:**
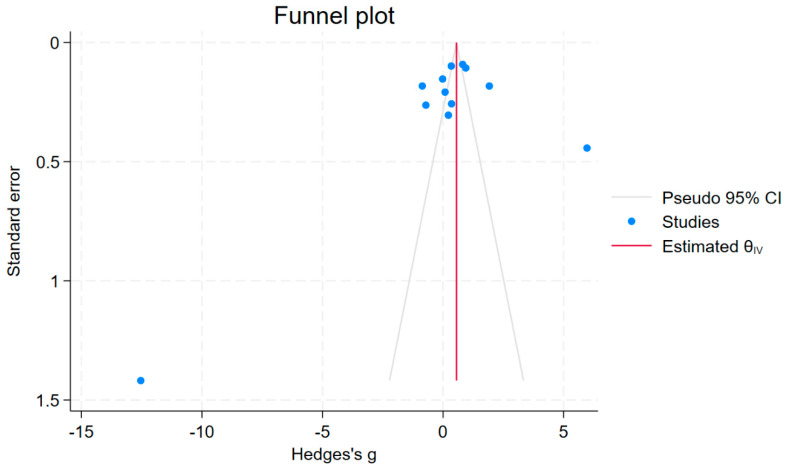
The funnel plot displays an asymmetrical distribution of effect estimates (blue dots) across the middle line (red vertical line).

Publication bias was assessed by visually inspecting the funnel plot symmetry and using Egger’s test. The funnel plot displays an asymmetrical distribution of effect estimates across the middle line (Figure 4). Egger’s test indicated a significant small-study effect (*p* < 0.05), suggesting publication bias due to the absence of smaller studies. After trimming and filling to adjust for this bias, the results showed that NO levels increased in patients with malaria compared to uninfected controls (Hedge’s g: 0.559, 95% CI: 0.469–0.648). The assessment of publication bias in the meta-analysis to determine differences in NO levels between patients with severe and non-severe malaria was not performed due to the few studies included in the meta-analysis (less than 10).

### 3.8. Sensitivity Analysis

**Figure 5:**
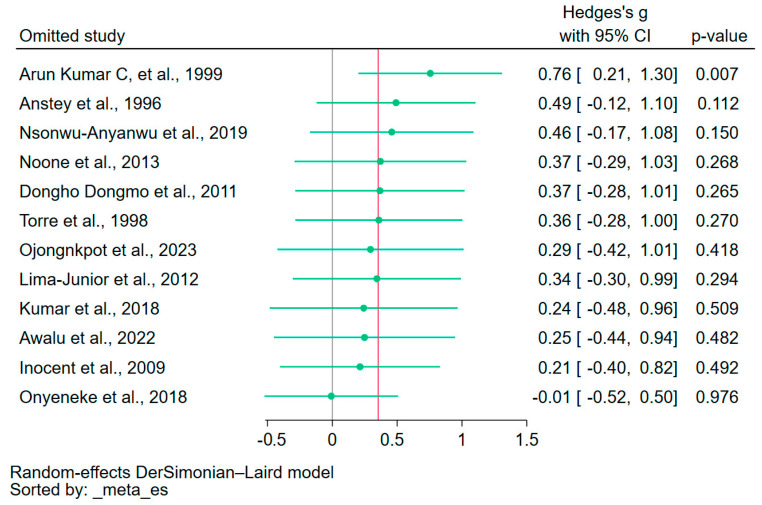
The leave-one-out analysis demonstrated significant influence from an individual study on the meta-analysis comparing patients with malaria to uninfected controls. The green dots indicate the pooled effect estimates for each rerun analysis. The red vertical line indicates the estimated overall results. The gray vertical line is the no-effect line. CI, confidence interval. References [16,30,31,35,37,39,40,42,43,44,46,47].

**Figure 6:**
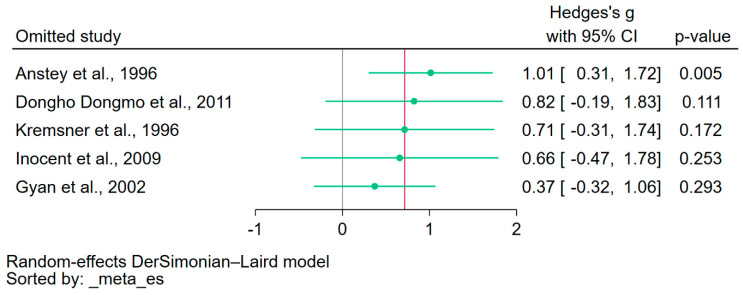
The leave-one-out analysis demonstrated significant influence from an individual study on the meta-analysis comparing patients with severe and non-severe malaria. The green dots indicate the pooled effect estimates for each rerun analysis. The red vertical line indicates the estimated overall results. The gray vertical line is the no-effect line. CI, confidence interval. References [16,35,36,37,38].

Leave-one-out analyses were performed for the meta-analyses to determine the differences in NO levels between patients with malaria and uninfected controls, and between patients with severe and non-severe malaria (Figure 5 and Figure 6, respectively). The analyses indicated a significant influence from an individual study in both comparisons, suggesting the meta-analysis results were not robust and could be unduly influenced by a single study in both comparisons [16,30].

## 4. Discussion

The results of this study elucidate the heterogeneous landscape of studies pertaining to the NO levels in malaria patients and uninfected controls. Several studies demonstrated higher NO levels in patients afflicted with malaria [31,33,39,40,44], especially patients manifesting symptoms [44] and experiencing severe forms of the disease, including severe malarial anemia [32,36]. Conversely, other studies reported decreased NO levels in malaria patients, especially uncomplicated and cerebral malaria cases [16,30,43]. However, a significant number of studies did not detect differences in the NO levels in patients with malaria compared with uninfected individuals [29,35,37,41,42,45,46,47]. In addition, several studies consistently found no significant differences in NO concentrations, irrespective of the severity or outcome of the falciparum malaria infection [29,34,35,37]. Nevertheless, a singular report showed elevated NO levels in asymptomatic malaria patients compared to asymptomatic uninfected individuals [23].

Although the thematic analysis revealed a variety of observations, no significant differences in mean NO levels between malaria patients and uninfected controls (*p* = 0.25) were detected in the meta-analysis, which was based on a considerable number of studies. The meta-analysis exhibited a high level of heterogeneity (*I*^2^: 97.39%). This heterogeneity prompted a more detailed analysis using meta-regression and subgroup analyses to determine the factors influencing the results, including publication years, geographic location, age group, NO measurement methods, and blood sample types. Recent studies (from 2020 to 2023) and studies conducted in Africa showed significant increases in the NO levels in malaria patients compared with uninfected patients. This trend was not observed in studies conducted before the year 2000 and studies conducted in Asia. A pressing question arises regarding the reasons behind the elevated NO levels observed in the more recent studies. However, only two recent studies were included in the subgroup meta-analysis; thus, the reasons for these discrepancies are unclear [31,44] but may be due to different methods of measuring the NO levels; the subgroup analysis highlighted a significant surge in NO levels in studies utilizing colorimetric methods to measure the NO levels [31,35,37,39]. 

Studies from Africa consistently showed elevated NO levels, a pattern not replicated in the Asian studies. Several factors may explain this disparity. Among the limited Asian-based studies, NO levels were notably inconsistent; one study highlighted a decrease in the NO levels in malaria patients [30], whereas another study indicated an increase in NO levels in patients with malaria [39]. While the dominant trend in the African studies was increased NO levels in patients with malaria, individual study results varied. Specifically, four studies deviated from the meta-analysis findings: two showed a reduction in NO levels [16,43], while the other two found no significant differences in NO levels [35,42]. In line with the Asian studies, studies from Europe [33] and South America [40] found no differences in the NO levels between malaria patients and uninfected controls. However, only two studies from these regions compared NO levels between the two groups. Thus, the evidence is not robust enough to draw conclusions, underscoring the need for additional research. 

A noteworthy demographic finding from the meta-analysis was the significant elevation of NO levels in adults compared to children. This discrepancy might stem from physiological differences, immune responses, or environmental influences [48,49]. Several studies found no differences in the NO levels in adults with malaria compared with noninfected controls, highlighting the necessity for further research to elucidate the role of NO levels in this demographic. The subgroup analysis assessing the impact of *Plasmodium* species on NO levels revealed that studies involving patients with *P. falciparum* malaria exhibited NO levels comparable to those of uninfected controls, indicating that fluctuations in NO levels were not necessarily associated with the species of *Plasmodium* present. Additional subgroup analyses that focused on symptomatology, NO measurement techniques, and blood sample types identified considerable variations. Specifically, certain methods and the use of serum samples were linked to significant augmentations in NO levels, emphasizing the influence these variables have on the results, and underscoring the complexity of assessing NO levels in malaria patients. The meta-analysis, including only the five studies examining disease severity, found no significant differences in the mean NO levels between severe and non-severe malaria cases (*p* = 0.09). Although a high degree of heterogeneity (*I*^2^: 96.07%) was noted in this subgroup analysis, severe *Plasmodium* infection did not markedly alter the NO levels compared to non-severe forms.

The reasons for the lack of differences in NO levels in the meta-analyses between malaria patients and uninfected individuals or between severe and non-severe malaria cases are unclear. In vitro studies demonstrated that the combination of NO and chloroquine reduces parasitemia in chloroquine-resistant malarial infections [50]. Exogenous nitric oxide from sodium nitroprusside effectively eradicates blood-stage *P. vivax* parasites in the laboratory setting [51]. Iron chelation therapy with desferrioxamine (DFO) before cytokine treatment increases NO production and enhances parasite destruction [52]. NO is a major effector molecule in the natural transmission-blocking mechanism against the malaria parasite by directly inhibiting the exflagellation of male gametocytes in *P. yoelii*-infected mice [53]. As for the relationship between disease severity and NO levels, in vitro studies using peripheral blood mononuclear cells show that patients with mild malaria exhibit significantly higher levels of NOS activity and NO production compared to individuals with severe malaria. This suggests that NOS/NO may be markers for prior disease severity and are key determinants of malaria resistance [54]. NO may also protect against severe *P. falciparum* malaria by inhibiting cytoadherence [55].

Variations in NO levels and NOS expression occur worldwide due to a combination of genetic, dietary, environmental, and disease-related factors. Specific polymorphisms in NOS genes can influence their expression and activity, leading to different NO production levels among global populations [56,57,58]. Dietary practices also play a role in variable NO levels; for instance, diets rich in nitrates from foods like beets and leafy greens can elevate NO levels [59,60]. Additionally, populations residing at higher altitudes may have increased NO production due to reduced oxygen availability; NO aids in oxygen delivery and vascular function [61,62]. Environmental factors, such as exposure to pollutants from cigarette smoke or industrial emissions, can modulate NO production and are more pronounced in heavily industrialized areas [63,64]. Disease prevalence, which varies by region, can also impact NO levels; conditions like cardiovascular diseases have been linked to altered NO production [65]. NO production can be altered in infectious diseases such as viral infections [12], bacterial infections [66], fungal infections [67], and parasitic infections [68]. Lastly, age-related changes in NO production and the varying age demographics across regions can influence overall NO levels [69,70]. 

Severe malaria is associated with reduced NO production and lower plasma L-arginine levels [71]. L-arginine supplements in patients with severe malaria can boost NO availability and improve outcomes [72]. Additionally, administering L-arginine may aid in the recovery of endothelial function in severe malaria cases accompanied by lactic acidosis. Given its potential to enhance endothelial NO production and overall endothelial function, L-arginine may be a beneficial adjunctive therapy in the early stages of severe malaria [73]. In addition to L-arginine, inhaled NO is an adjunctive treatment for severe malaria, including cerebral malaria. Inhaled NO improves neurocognitive outcomes and lactic acidosis [74], and enhances endothelial NO bioavailability [75].

This systematic review and meta-analysis present several limitations. A high degree of heterogeneity, as demonstrated by *I*^2^ values exceeding 96%, was encountered in both meta-analyses, highlighting the considerable variability in the outcomes of the incorporated studies. This variability may undermine the reliability of the pooled results. The range of methodologies employed across the studies, encompassing different diagnostic approaches, NO measurement techniques, and blood sample types, introduces another layer of complexity and potential source of bias, hindering the derivation of unified conclusions. Moreover, the scarce representation of recent studies, particularly those published between 2020 and 2023, raises questions regarding the current relevance of the findings, given the evolving dynamics of malaria epidemiology and advances in diagnostic techniques. The subgroup analyses, vital for a nuanced understanding of the data, were constrained by the limited data, which was especially apparent in the evaluation of NO levels between severe and non-severe malaria cases. Additionally, the review noted significant geographical and demographic disparities in the NO levels, without a thorough exploration of the underlying causes, leaving a crucial gap in the interpretation of results. 

This systematic review and meta-analysis underscore the need for further research into the relationship between NO and malaria. Inconsistencies and heterogeneity were observed across the studies included in the analysis. While no significant differences in NO levels were detected between various groups, the role of NO in malaria remains a topic of interest. Future studies should adopt a standardized approach for measuring NO to ensure consistency across studies. Moreover, delving deeper into the biochemical pathways that govern NO responses to malaria may offer valuable insights into therapeutic advances. Determining NO levels in patients, when combined with other metrics and harmonized conditions, may be instrumental in understanding the association between NO and malaria.

## 5. Conclusions

This systematic review and meta-analysis revealed inconsistent NO levels among patients with malaria. Overall, the results indicate no significant differences in the NO levels between patients with malaria and uninfected controls, or between patients with severe and non-severe malaria. As evidenced by a marked increase in recent studies showing elevated NO levels in malaria patients, especially in Africa and in studies utilizing specific NO measurement techniques and blood sample types, a directional shift in understanding the role of NO in malaria physiology necessitates further exploration with refined methodology. Further research exploring the biochemical pathways governing NO responses in malaria and how these responses can be leveraged for therapeutic advancements is warranted.

## Figures and Tables

**Table 1:** Summary characteristics of studies.

Characteristics	*n*. (21 Studies)	%
**Publication year**		
Before 2000	7	33.33
2000–2009	4	19.05
2010–2019	8	38.10
2020–2023	2	9.52
**Study designs**		
Case–control studies	7	33.33
Cross-sectional studies	9	42.86
Cohort studies	5	23.81
**Study areas**		
Asia	3	14.29
- India	2	9.52
- Thailand	1	4.76
Africa	14	66.67
- Nigeria	4	19.05
- Cameroon	4	19.05
- Ghana	3	14.29
- Tanzania	2	9.52
- Gabon	1	4.76
South America (Brazil)	2	9.52
Europe	2	9.52
- Portugal	1	4.76
- Italy	1	4.76
***Plasmodium* spp** .		
*P. falciparum*	16	76.19
*P. falciparum*, *P. vivax*	2	9.52
*P. falciparum*, *P. vivax*, *P. malariae*, *P. ovale*	1	4.76
*P. falciparum*, *P. vivax*, mixed infections	1	4.76
Not specified	1	4.76
**Participants**		
Children	9	42.86
Adults	9	42.86
Children and adults	2	9.52
Not specified	1	4.76
**Symptom**		
Symptomatic	14	66.67
Asymptomatic	1	4.76
Symptomatic and asymptomatic malaria	1	4.76
Not specified	5	23.81
**Severity status**		
Severe and uncomplicated	6	28.57
Non-severe malaria (uncomplicated or asymptomatic malaria)	5	23.81
Severe malaria	2	9.52
Asymptomatic	1	4.76
Not specified	7	3.33
**Methods for malaria detection**		
Microscopic method	11	52.38
Microscopic method, PCR	4	19.02
Microscopic method, RDT	2	9.52
Microscopic method, RDT, PCR	1	4.76
Not specified	3	14.29
**Method for NO**		
Griess assay	12	57.14
Colorimetric method (not specified assay)	4	19.05
Capillary electrophoresis	3	14.29
Ion-pair chromatography	1	4.76
Not specified	1	4.76
**Blood sample for NO measurement**		
Plasma	12	57.14
Serum	6	28.57
Not specified	3	14.29

Abbreviations: PCR, polymerase chain reaction; RDT, rapid diagnostic test; NO, nitric oxide.

**Table 2:** Comparison of nitric oxide levels in patients with malaria.

No.	Authors	Study Location	*Plasmodium* spp.	Age Range (Years)	NO Levels in Patients with Malaria
1.	Agbenyega et al., 1997 [29]	Ghana	*P. falciparum*	Children	1. No difference in NO concentration between malaria and uninfected controls. 2. No difference in NO concentration between severe and non-severe malaria.
2.	Anstey et al., 1996 [16]	Tanzania	*P. falciparum*	6 months–9 years	1. NOx was significantly lower in those with UM and CM than uninfected controls. 2. NOx was significantly higher in asymptomatic malaria than uninfected controls, and CM.
3.	Anstey et al., 1999 [23]	Tanzania	*P. falciparum*	6 months–9 years	NO was significantly higher in asymptomatic malaria (thick film positive) than asymptomatic malaria (thick film negative/PCR positive) and uninfected controls (thick film negative/PCR negative).
4.	Arun Kumar C and Das UN [30]	India	*P. falciparum*	Children and Adults	NO levels were significantly lower in patients with malaria as compared to uninfected controls.
5.	Awalu et al., 2022 [31]	Nigeria	Not specified	17–21 years	NO levels were significantly higher in patients with malaria as compared to uninfected controls.
6.	Cramer et al., 2005 [32]	Ghana	*P. falciparum*	6 months–9 years	NO levels were significantly higher in patients with severe malarial anemia than in children without malaria.
7.	De Sousa et al., 2008 [33]	Portugal	*P. falciparum*	Not specified	NO levels were significantly higher in patients with malaria than in children without malaria.
8.	Dondorp et al., 1998 [34]	Thailand	*P. falciparum*	Not specified	No difference in NO concentration between survivors and fatal cases of malaria.
9.	Dongho Dongmo et al., 2011 [35]	Cameroon	*P. falciparum*	≤15 years	No difference in NO concentration between cerebral malaria, malaria anemia, uncomplicated malaria, and controls.
10.	Gyan et al., 2002 [36]	Ghana	*P. falciparum*	0.5–12 years	NO levels were significantly higher in patients with severe anemia as compared to cerebral malaria and uncomplicated malaria.
11.	Inocent et al., 2009 [37]	Cameroon	*P. falciparum*	0–15 years	1. No difference in NO concentration between malaria and uninfected controls. 2. No difference in NO concentration between severe (cerebral malaria, cerebral malaria with malarial anemia, malarial anemia) and non-severe malaria.
12.	Kremsner et al., 1996 [38]	Gabon	*P. falciparum*	Children and Adults	NO levels were significantly higher in patients with severe malaria as compared to uncomplicated malaria.
13.	Kumar et al., 2018 [39]	India	*P. falciparum*, *P. vivax*	Adult	NOx levels were significantly higher in patients with malaria (*P. falciparum* and *P. vivax*) as compared to controls.
14.	Lima-Junior et al., 2012 [40]	Brazil	*P. falciparum*, *P. vivax*	Not specified	NO levels were significantly higher in patients with *P. falciparum* malaria as compared to controls.
15.	Megnekou et al., 2015 [41]	Cameroon	*P. falciparum*	16–39 years	No difference in NO concentration between malaria and uninfected controls.
16.	Noone et al., 2013 [42]	Nigeria	*P. falciparum*	39–73 months	No difference in NO concentration between malaria and uninfected controls.
17.	Nsonwu-Anyanwu et al., 2019 [43]	Nigeria	*P. falciparum*	18–60 years	NO levels were significantly lower in patients with malaria as compared to uninfected controls.
18.	Ojongnkpot et al., 2023 [44]	Cameroon	*P. falciparum*	1–15 years	1. NO levels were significantly higher in patients with malaria as compared to uninfected controls. 2. NO levels were significantly higher in patients with symptomatic malaria as compared to asymptomatic malaria.
19.	Onyeneke et al., 2018 [47]	Nigeria	*P. falciparum*	Not specified	No difference in NO concentration between malaria and uninfected controls.
20.	Sánchez-Arcila et al., 2014 [45]	Brazil	*P. falciparum*, *P. vivax*, mixed infections	14–38 years	No difference in NO concentration between malaria and uninfected controls.
21.	Torre et al., 1998 [46]	Italy	*P. falciparum*, *P. vivax*, *P. malariae*, *P. ovale*	18–44 years	No difference in NO concentration between malaria and uninfected controls.

Abbreviations: NO, nitric oxide; NOx, nitrogen oxides; UM, uncomplicated malaria, CM, cerebral malaria.

**Table 3:** Subgroup analyses of NO levels between patients with malaria and uninfected controls.

Subgroup Analyses	*p*-Value	Hedges’ g (95% CI)	*I*^2^ (%)	Number of Studies
Publication years				
2020–2023	0.03	0.64 (0.05–1.23)	94.10	2
2010–2019	0.04	1.01 (0.04–1.97)	97.51	6
2000–2009	N/A	1.92 (1.56–2.28)	N/A	1
Before 2000	0.01	−3.52 (−6.11–(−0.93))	97.49	3
Study design				
Cross-sectional study	0.74	0.09 (−0.43–0.60)	94.80	5
Case-control study	0.44	−1.43 (−5.06–2.21)	98.84	4
Cohort study		1.09 (−5.06–2.21)		3
Continent				
Africa	0.02	0.89 (0.12–1.66)	97.85	8
Asia	0.39	−5.79 (−18.87–7.30)	98.87	2
Europe	N/A	0.22 (−0.38–0.82)	N/A	1
South America	N/A	0.35 (−0.15–0.86)	N/A	1
Age group				
Children	0.45	0.29 (−0.47–1.06)	96.80	5
Adults	0.01	1.18 (0.33–2.03)	97.19	6
Children and adults	N/A	−12.54 (−15.32–(−9.76))	N/A	1
*Plasmodium* species				
*P. falciparum*	0.99	−0.01 (−1.04–1.02)	98.18	8
*P. falciparum*, *P. vivax*	<0.01	0.65 (0.21–1.08)	65.12	2
*P. falciparum*, *P. vivax*, *P. malariae*, *P. ovale*	N/A	0.22 (−0.38–0.82)	N/A	1
Not specified	N/A	0.94 (0.73–1.15)	N/A	1
Symptoms				
Symptomatic	0.90	−0.04 (−0.67–0.59)	96.70	9
Symptomatic and asymptomatic malaria	N/A	0.34 (0.15–0.53)	N/A	1
Not specified	0.43	2.62 (−3.93–9.17)	99.41	2
Severity status				
Severe and uncomplicated malaria	0.65	0.38 (−1.29–2.05)	98.33	3
Non-severe malaria (uncomplicated or asymptomatic malaria)	0.04	0.36 (0.02–0.70)	85.01	5
Not specified	0.47	−1.02 (−3.79–1.75)	98.84	4
Methods for malaria detection				
Microscopic method	0.60	−0.29 (−1.36–0.79)	97.99	7
Microscopic method, PCR	N/A	0.35 (−0.15–0.86)	N/A	1
Microscopic method, RDT	N/A	1.92 (1.56–2.28)	N/A	1
Microscopic method, RDT, PCR	N/A	0.81 (0.63–1.00)	N/A	1
Not specified	0.07	0.64 (−0.06–1.33)	N/A	2
Method for NO				
Griess assay	0.09	−0.67 (−1.45–0.11)	94.81	6
Colorimetric method (not specified assay)	<0.01	0.94 (0.42–1.47)	93.69	4
Capillary electrophoresis	N/A	0.86 (−1.22–(−0.50))	N/A	1
Ion-pair chromatography				
Not specified	N/A	5.97 (5.10–6.84)	N/A	1
Types of blood samples				
Serum	0.01	1.23 (0.24–2.22)	97.85	5
Plasma	0.10	−1.20 (−2.63–0.22)	98.05	5
Not specified	0.22	0.53 (−0.31–1.37)	92.56	2

Abbreviations: CI, confidence interval; N/A, not assessed; NO, nitric oxide; PCR, polymerase chain reaction; RDT, rapid diagnostic test.

## Data Availability

Data is contained within the article and supplementary materials.

## Supplementary Materials

The following supporting information can be downloaded at: https://www.mdpi.com/article/10.3390/antiox12101868/s1, Table S1: Search terms; Table S2: Details of included studies; Table S3: Quality of studies; Table S4: Meta-regression analyses; PRISMA Abstract Checklist; PRISMA 2020 Checklist.

## References

[1] WHO (2022). World Malaria Report 2022.

[2] Menkin-Smith L., Winders W.T. (2023). Plasmodium vivax malaria.

[3] Zekar L., Sharman T. (2023). Plasmodium falciparum malaria.

[4] (2021). Malaria: (Still) A global health priority. EClinicalMedicine.

[5] Garcia X., Stein F. (2006). Nitric oxide. Semin. Pediatr. Infect. Dis..

[6] Bruckdorfer R. (2005). The basics about nitric oxide. Mol. Asp. Med..

[7] Lundberg J.O., Weitzberg E. (2022). Nitric oxide signaling in health and disease. Cell.

[8] Gantner B.N., LaFond K.M., Bonini M.G. (2020). Nitric oxide in cellular adaptation and disease. Redox Biol..

[9] Lundberg J.O., Weitzberg E., Gladwin M.T. (2008). The nitrate-nitrite-nitric oxide pathway in physiology and therapeutics. Nat. Rev. Drug Discov..

[10] Andres C.M.C., Perez de la Lastra J.M., Juan C.A., Plou F.J., Perez-Lebena E. (2022). The role of reactive species on innate immunity. Vaccines.

[11] Kleinert H., Forstermann U., Enna S.J., Bylund D.B. (2007). Inducible nitric oxide synthase. xPharm: The Comprehensive Pharmacology Reference.

[12] Akaike T., Maeda H. (2000). Nitric oxide and virus infection. Immunology.

[13] Bath P.M., Coleman C.M., Gordon A.L., Lim W.S., Webb A.J. (2021). Nitric oxide for the prevention and treatment of viral, bacterial, protozoal and fungal infections. F1000Research.

[14] Pavanelli W.R., Gutierrez F.R.S., da Silva J.J.N., Costa I.C., de Menezes M.C.N.D., de Abreu Oliveira F.J., Itano E.N., Watanabe M.A.E. (2010). The effects of nitric oxide on the immune response during giardiasis. Braz. J. Infect. Dis..

[15] Jones-Carson J., Yahashiri A., Kim J.-S., Liu L., Fitzsimmons L.F., Weiss D.S., Vázquez-Torres A. (2020). Nitric oxide disrupts bacterial cytokinesis by poisoning purine metabolism. Sci. Adv..

[16] Anstey N.M., Weinberg J.B., Hassanali M.Y., Mwaikambo E.D., Manyenga D., Misukonis M.A., Arnelle D.R., Hollis D., McDonald M.I., Granger D.L. (1996). Nitric oxide in Tanza-nian children with malaria: Inverse relationship between malaria severity and nitric oxide production/nitric oxide synthase type 2 expression. J. Exp. Med..

[17] Boutlis C.S., Tjitra E., Maniboey H., Misukonis M.A., Saunders J.R., Suprianto S., Weinberg J.B., Anstey N.M. (2003). Nitric oxide production and mono-nuclear cell nitric oxide synthase activity in malaria-tolerant Papuan adults. Infect. Immun..

[18] A Wink D., Hines H.B., Cheng R.Y.S., Switzer C.H., Flores-Santana W., Vitek M.P., A Ridnour L., A Colton C. (2011). Nitric oxide and redox mechanisms in the immune response. J. Leukoc. Biol..

[19] Tripathi P. (2007). Nitric oxide and immune response. Indian. J. Biochem. Biophys..

[20] Pierini D., Bryan N.S. (2015). Nitric oxide availability as a marker of oxidative stress. Methods Mol. Biol..

[21] James S.L. (1995). Role of nitric oxide in parasitic infections. Microbiol. Rev..

[22] Hobbs M.R., Udhayakumar V., Levesque M.C., Booth J., Roberts J.M., Tkachuk A.N., Pole A., Coon H., Kariuki S., Nahlen B.L. (2002). A new NOS2 promoter polymorphism associated with increased nitric oxide production and protection from severe malaria in Tanzanian and Kenyan children. Lancet.

[23] Anstey N.M., Mwaikambo E.D., Wang Z., Granger D.L., E Duffy P., Weinberg J.B. (1999). Effects of age and parasitemia on nitric oxide production/leukocyte nitric oxide synthase type 2 expression in asymptomatic, malaria-exposed children. Am. J. Trop. Med. Hyg..

[24] Page M.J., McKenzie J.E., Bossuyt P.M., Boutron I., Hoffmann T.C., Mulrow C.D., Shamseer L., Tetzlaff J.M., Akl E.A., Brennan S.E. (2021). The PRISMA 2020 statement: An updated guideline for reporting systematic reviews. BMJ.

[25] Morgan R.L., Whaley P., Thayer K.A., Schunemann H.J. (2018). Identifying the PECO: A framework for formulating good questions to explore the association of environmental and other exposures with health outcomes. Environ. Int..

[26] Moola S., Munn Z., Tufanaru C., Aromataris E., Sears K., Sfetcu R., Currie M., Qureshi R., Mattis P., Lisy K. (2020). Chapter 7: Systematic Reviews of Etiology and Risk.

[27] Thomas J., Harden A. (2008). Methods for the thematic synthesis of qualitative research in systematic reviews. BMC Med. Res. Methodol..

[28] Higgins J.P.T., Thompson S.G. (2002). Quantifying heterogeneity in a meta-analysis. Stat. Med..

[29] Agbenyega T., Angus B., Bedu-Addo G., Baffoe-Bonnie B., Griffin G., Vallance P., Krishna S. (1997). Plasma nitrogen oxides and blood lactate concentrations in Ghanaian children with malaria. Trans. R. Soc. Trop. Med. Hyg..

[30] Arun Kumar C., Das U.N. (1999). Lipid peroxides, nitrate oxide and essential fatty acids in patients with *Plasmodium falciparum* malaria. Prostaglandins Leukot. Essent. Fatty Acids.

[31] Awalu J.C., Ukibe N.R., Onyenekwe C.C., Ahaneku J.E., Ihim A.C., Udeh T., Onah C.E., Ehiaghe F.A., Ukibe G.E., Ukibe B.C. (2022). Assessment of oxidative stress and antioxidant status in newly admitted healthy undergraduate students in Nnamdi Azi-kiwe University Awka, Nigeria. J. Pharm. Negat. Results.

[32] Cramer J.P., Nüssler A.K., Ehrhardt S., Burkhardt J., Otchwemah R.N., Zanger P., Dietz E., Gellert S., Bienzle U., Mockenhaupt F.P. (2005). Age-dependent effect of plasma nitric oxide on parasite density in Ghanaian children with severe malaria. Trop. Med. Int. Health.

[33] De Sousa K., Silva M.S., Tavira L.T. (2008). Variation of nitric oxide levels in imported *Plasmodium falciparum* malaria episodes. Afr. J. Biotechnol..

[34] Dondorp A.M., Vreeken J., Planche T., Chotivanich K.T., White N.J., Ruangveerayuth R., Silamut K., E de Bel E., A Romijn J., A Kager P. (1998). Nitric oxides in plasma, urine, and cerebrospinal fluid in patients with severe falciparum malaria. Am. J. Trop. Med. Hyg..

[35] Dongmo F.D., Ngane R.N., Gouado I., Mfonkeu J.P., Kwemba V.M., Ngwa V., Kuate H.F., Zollo P.A. (2011). Predictors of childhood severe malaria in a densely populated area: Douala, cameroon. Afr. J. Biotechnol..

[36] Gyan B., Kurtzhals J.A., Akanmori B.D., Ofori M., Goka B.Q., Hviid L., Behr C. (2002). Elevated levels of nitric oxide and low levels of haptoglobin are associated with severe malarial anaemia in African children. Acta Trop..

[37] Inocent G., Bertrand P.M.J., Honoré F.K., Odette Z., Salomé N., Valéry C., Georges G.E., Henri A.Z.P. (2009). Physiopathologic factors resulting in poor outcome in childhood severe malaria in Cameroon. Pediatr. Infect. Dis. J..

[38] Kremsner P.G., Winkler S., Wildling E., Prada J., Bienzle U., Graninger W., Nüssler A.K. (1996). High plasma levels of nitrogen oxides are associated with severe disease and correlate with rapid parasitological and clinical cure in *Plasmodium falciparum* malaria. Trans. R. Soc. Trop. Med. Hyg..

[39] Kumar A., Singh K.P., Bali P., Anwar S., Kaul A., Singh O.P., Gupta B.K., Kumari N., Alam N., Raziuddin M. (2018). iNOS polymorphism modulates iNOS/NO expression via impaired antioxidant and ROS content in *P. vivax* and *P. falciparum* infection. Redox Biol..

[40] Lima-Junior J.D.C., Rodrigues-da-Silva R.N., Pereira V.A., Storer F.L., Perce-da-Silva D.D.S., Fabrino D.L., Santos F., Banic D.M., Oliveira-Ferreira J.D. (2012). Cells and mediators of inflammation (C-reactive protein, nitric oxide, platelets and neutrophils) in the acute and convalescent phases of un-complicated *Plasmodium vivax* and *Plasmodium falciparum* infection. Mem. Inst. Oswaldo Cruz..

[41] Megnekou R., Djontu J.C., Bigoga J.D., Medou F.M., Tenou S., Lissom A. (2015). Impact of placental *Plasmodium falciparum* malaria on the profile of some oxidative stress biomarkers in women living in Yaoundé, Cameroon. PLoS ONE.

[42] Noone C., Parkinson M., Dowling D.J., Aldridge A., Kirwan P., Molloy S.F., Asaolu S.O., Holland C., O’Neill S.M. (2013). Plasma cytokines, chemokines and cellular immune responses in pre-school Nigerian children infected with *Plasmodium falciparum*. Malar. J..

[43] Nsonwu-Anyanwu A.C., Osuoha U.O., Nsonwu M.C., Usoro C.A.O. (2019). Antimalaria therapy and changes in oxidative stress indices in falciparum malaria infection in Calabar metropolis, Nigeria. Trop. J. Pharm. Res..

[44] Ojongnkpot T.A., Sofeu-Feugaing D.D., Jugha V.T., Taiwe G.S., Kimbi H.K. (2023). implication of oxidative stress and antioxidant defence systems in symptomatic and asymptomatic *Plasmodium falciparum* malaria infection among children aged 1 to 15 years in the Mount Cameroon Area. J. Biosci. Med..

[45] Sánchez-Arcila J.C., Perce-da-Silva D.D.S., Vasconcelos M.P.A., Rodrigues-da-Silva R.N., Pereira V.A., Aprígio C.J.L., Lima C.A.M., Banic D.M., Lima-Junior J.D.C., Oliveira-Ferreira J. (2014). Intestinal parasites coinfection does not alter plasma cytokines profile elicited in acute malaria in subjects from endemic area of Brazil. Mediat. Inflamm..

[46] Torre D., Ferrario G., Matteelli A., Speranza F., Giola M., Pugliese A., Cantamessa C., Carosi G., Fiori G.P. (1998). Levels of circulating nitrate/nitrite and gamma interferon not increased in uncomplicated malaria. Infection.

[47] Onyeneke E.C.O.P., Anyanwu G.O., Onovughakpo-Sakpa E.O., Anionye J.C., Anekwe A.I. (2018). Evaluation of nitric oxide and antioxidant status of *Plasmodium falciparum* infected pregnant Nigerian women with malaria. Idosr J. Sci. Res..

[48] Long C.A., Zavala F. (2017). Immune Responses in Malaria. Cold Spring Harb. Perspect. Med..

[49] Nkuo-Akenji T., Ntonifor N.N., Ndukum M.B., Kimbi H.K., Abongwa E.L., Nkwescheu A., Anong D.N., Songmbe M., Boyo M.G., Ndamukong K.N. (2006). Environmental factors affecting malaria parasite prevalence in rural Bolifamba, South West Cameroon. Afr. J. Health Sci..

[50] Awasthi A., Kumar A., Upadhyay S.N., Yamada T., Matsunaga Y. (2003). Nitric oxide protects against chloroquine resistant *Plas-modium yoelii nigeriensis* parasites in vitro. Exp. Parasitol..

[51] Fang Q., Xia H., Wang X.M., Lu F., Tao Z.Y., Cao J., Sun X., Gao Q. (2009). In vitro lethal effect of exogenous nitric oxide on blood-stage *Plasmodium vivax*. Zhongguo Ji Sheng Chong Xue Yu Ji Sheng Chong Bing Za Zhi.

[52] Fritsche G., Larcher C., Schennach H., Weiss G. (2001). Regulatory interactions between iron and nitric oxide metabolism for immune defense against *Plasmodium falciparum* infection. J. Infect. Dis..

[53] Liu Y.-J., Wang J.-C., Feng H., Zhu X.-T., An C.-L., Cao Y.-M. (2007). In vitro observation on effect of nitric oxide on exflagellation of *Plasmodium yoelii*. Zhongguo Ji Sheng Chong Xue Yu Ji Sheng Chong Bing Za Zhi = Chin. J. Parasitol. Parasit. Dis..

[54] Perkins D.J., Kremsner P.G., Schmid D., Misukonis M.A., Kelly M.A., Weinberg J.B. (1999). Blood mononuclear cell nitric oxide production and plasma cytokine levels in healthy Gabonese children with prior mild or severe malaria. Infect. Immun..

[55] Serirom S., Raharjo W.H., Chotivanich K., Loareesuwan S., Kubes P., Ho M. (2003). Anti-adhesive effect of nitric oxide on *Plasmodium falciparum* cytoadherence under flow. Am. J. Pathol..

[56] Yadav S.K., Gupta S., Yadav A., Bhatt M.L., Mishra D.P., Roy D., Sanyal S. (2019). Endothelial nitric oxide synthase gene polymorphisms modulate the risk of squamous cell carcinoma of head and neck in north Indian population. Meta Gene.

[57] Qidwai T., Jamal F. (2010). Inducible nitric oxide synthase (iNOS) gene polymorphism and disease prevalence. Scand. J. Immunol..

[58] Dhangadamajhi G., Mohapatra B.N., Kar S.K., Ranjit M. (2009). Endothelial nitric oxide synthase gene polymorphisms and *Plasmodium falciparum* infection in Indian adults. Infect. Immun..

[59] Sweazea K.L., Johnston C.S., Miller B., Gumpricht E. (2018). Nitrate-rich fruit and vegetable supplement reduces blood pressure in normotensive healthy young males without significantly altering flow-mediated vasodilation: A randomized, double-blinded, controlled trial. J. Nutr. Metab..

[60] Lidder S., Webb A.J. (2013). Vascular effects of dietary nitrate (as found in green leafy vegetables and beetroot) via the nitrate-nitrite-nitric oxide pathway. Br. J. Clin. Pharmacol..

[61] Beall C.M., Laskowski D., Erzurum S.C. (2012). Nitric oxide in adaptation to altitude. Free Radic. Biol. Med..

[62] Feelisch M. (2018). Enhanced nitric oxide production is a universal response to hypoxic stress. Natl. Sci. Rev..

[63] Braun M., Klingelhöfer D., Müller R., Groneberg D.A. (2021). The impact of second-hand smoke on nitrogen oxides concentrations in a small interior. Sci. Rep..

[64] Wei X.M., Kim H.S., Kumar R.K., Heywood G.J., E Hunt J., McNeil H.P., Thomas P.S. (2005). Effects of cigarette smoke on degranulation and NO production by mast cells and epithelial cells. Respir. Res..

[65] Adam L.N., Oraha A.Y., Shekha M.S., Al-Habib O.A. (2023). Exploring nitric oxide as a crucial prognostic biomarker of coronary artery disease. Prostaglandins Other Lipid Mediat..

[66] Such J., Francés R., Pérez-Mateo M. (2002). Nitric oxide in patients with cirrhosis and bacterial infections. Metab. Brain Dis..

[67] Tsuji S., Taniuchi S., Hasui M., Yamamoto A., Kobayashi Y. (2002). Increased nitric oxide production by neutrophils from patients with chronic granulomatous disease on trimethoprim–sulfamethoxazole. Nitric Oxide.

[68] Brunet L.R. (2001). Nitric oxide in parasitic infections. Int. Immunopharmacol..

[69] Toprakçı M., Özmen D., Mutaf I., Turgan N., Parıldar Z., Habif S., Güner I., Bayındır O. (2000). Age-associated changes in nitric oxide metabolites nitrite and nitrate. Int. J. Clin. Lab. Res..

[70] Kawamoto E.M., Vasconcelos A.R., Degaspari S., Bohmer A.E., Scavone C., Marcourakis T. (2013). Age-related changes in nitric oxide activity, cyclic GMP, and TBARS levels in platelets and erythrocytes reflect the oxidative status in central nervous sys-tem. Age.

[71] Weinberg J.B., Lopansri B.K., Mwaikambo E., Granger D.L. (2008). Arginine, nitric oxide, carbon monoxide, and endothelial function in severe malaria. Curr. Opin. Infect. Dis..

[72] Yeo T.W., Rooslamiati I., Gitawati R., Tjitra E., Lampah D.A., Kenangalem E., McNeil Y.R., Price R.N., Anstey N.M., Duffull S.B. (2008). Pharmacokinetics of l -Arginine in adults with moderately severe malaria. Antimicrob. Agents Chemother..

[73] Yeo T.W., Lampah D.A., Gitawati R., Tjitra E., Kenangalem E., McNeil Y.R., Darcy C.J., Granger D.L., Weinberg J.B., Lopansri B.K. (2008). Recovery of endothelial function in severe falciparum malaria: Relationship with improvement in plasma L-arginine and blood lactate concentrations. J. Infect. Dis..

[74] Bangirana P., Conroy A.L., Opoka R.O., Hawkes M., Hermann L., Miller C., Namasopo S., Liles C.W., John C.C., Kain K.C. (2016). Inhaled nitric oxide improves neurocognitive outcomes in children with severe malaria and lactic acidosis. Am. J. Trop. Med. Hyg..

[75] Hawkes M.T., Conroy A.L., Opoka R.O., Hermann L., Thorpe K.E., McDonald C., Kim H., Higgins S., Namasopo S., John C. (2015). Inhaled nitric oxide as adjunctive therapy for severe malaria: A randomized controlled trial. Malar. J..

