# Supplementary material for: Association between Plasmodium Infection and Nitric Oxide Levels: A Systematic Review and Meta-Analysis
Source: Antioxidants (Basel). 2023 Oct 16;12(10):1868. doi: 10.3390/antiox12101868 (PMC10604424; doi:10.3390/antiox12101868)
Supplement: Supplementary file 1 [file antioxidants-12-01868-s001.zip › Table S1. Search terms.pdf]

Association between nitric oxide levels and status of *Plasmodium* infection: A systematic review and meta-analysis

Kwuntida Uthaisar Kotepui[1†], Aongart Mahittikorn[2†], Polrat Wilairatana[3*], Frederick Ramirez Masangkay[4], Manas Kotepui[1]*

[1]Medical Technology, School of Allied Health Sciences, Walailak University, Thasala, Nakhon Si Thammarat 80160, Thailand

[2]Department of Protozoology, Faculty of Tropical Medicine, Mahidol University, Bangkok 10400, Thailand

[3]Department of Clinical Tropical Medicine, Faculty of Tropical Medicine, Mahidol University, Bangkok 10400, Thailand

[4]Department of Medical Technology, Faculty of Pharmacy, University of Santo Tomas, Manila 1000, Philippines

*Corresponding author

† These authors contributed equally to this work.

Kwuntida Uthaisar Kotepui: kwuntida.ut@wu.ac.th

Aongart Mahittikorn: aongart.mah@mahidol.ac.th

Frederick Ramirez Masangkay: frederick_masangkay2002@yahoo.com

Polrat Wilairatana: polrat.mah@mahidol.ac.th

Manas Kotepui manas.ko@wu.ac.th, Tel+ :.66954392469

Table S1. Search terms

MeSH terms

Nitric oxide: Nitrogen Monoxide; Endothelium-Derived Nitric Oxide; Endogenous Nitrate Vasodilator; Mononitrogen Monoxide

Malaria: plasmodium; Remittent Fever; Marsh Fever; Paludism

General keywords

(“Nitric oxide” OR “Nitrogen Monoxide” OR “Endothelium-Derived Nitric Oxide” OR “Endogenous Nitrate Vasodilator” OR “Mononitrogen Monoxide”) AND (malaria OR plasmodium OR “Plasmodium Infection“ OR “Remittent Fever“ OR “Marsh Fever“ OR Paludism)

PubMed 25 August 2023

No.	Key concept	Search terms	Results
1.	Nitric oxide	“Nitric oxide”[Text Word] OR “Nitric oxide”[MeSH Terms] OR “Nitrogen Monoxide”[Text Word] OR “Endothelium-Derived Nitric Oxide”[Text Word] OR “Endogenous Nitrate Vasodilator”[Text Word] OR “Mononitrogen Monoxide”[Text Word]	192,830
2.	Malaria	malaria[Text Word] OR plasmodium[Text Word] OR malaria[MeSH Terms] OR “Remittent Fever“[Text Word] OR “Marsh Fever“[Text Word] OR Paludism[Text Word]	119,191
3.	#1 AND #2	(“Nitric oxide”[Text Word] OR “Nitric oxide”[MeSH Terms] OR “Nitrogen Monoxide”[Text Word] OR “Endothelium-Derived Nitric Oxide”[Text Word] OR “Endogenous Nitrate Vasodilator”[Text Word] OR “Mononitrogen Monoxide”[Text Word]) AND (malaria[Text Word] OR plasmodium[Text Word] OR malaria[MeSH Terms] OR “Remittent Fever“[Text Word] OR “Marsh Fever“[Text Word] OR Paludism[Text Word])	658

Embase 25 August 2023

No.	Key concept	Search terms	Results
1.	Nitric oxide	'nitric oxide':ti,ab,kw,de OR 'nitric oxide'/exp OR 'nitrogen monoxide':ti,ab,kw,de OR 'endothelium-derived nitric oxide':ti,ab,kw,de OR 'endogenous nitrate vasodilator':ti,ab,kw,de OR 'mononitrogen monoxide':ti,ab,kw,de	294,962
2.	Malaria	malaria:ti,ab,kw,de OR plasmodium:ti,ab,kw,de OR 'remittent fever':ti,ab,kw,de OR 'marsh fever':ti,ab,kw,de OR paludism:ti,ab,kw,de OR 'malaria'/exp	157,388

3.	#1 AND #2	('nitric oxide':ti,ab,kw,de OR 'nitric oxide'/exp OR 'nitrogen monoxide':ti,ab,kw,de OR 'endothelium-derived nitric oxide':ti,ab,kw,de OR 'endogenous nitrate vasodilator':ti,ab,kw,de OR 'mononitrogen monoxide':ti,ab,kw,de) AND (malaria:ti,ab,kw,de OR plasmodium:ti,ab,kw,de OR 'remittent fever':ti,ab,kw,de OR 'marsh fever':ti,ab,kw,de OR paludism:ti,ab,kw,de OR 'malaria'/exp)	1,234

Scopus 25 August 2023

No.	Key concept	Search terms	Results
1.	Nitric oxide	TITLE-ABS-KEY (“Nitric oxide” OR “Nitrogen Monoxide” OR “Endothelium-Derived Nitric Oxide” OR “Endogenous Nitrate Vasodilator” OR “Mononitrogen Monoxide”)	298,387
2.	Malaria	TITLE-ABS-KEY ((malaria OR plasmodium OR "plasmodium infection" OR "remittent fever" OR "marsh fever" OR paludism))	158,195
3.	1 AND 2	(“Nitric oxide” OR “Nitrogen Monoxide” OR “Endothelium-Derived Nitric Oxide” OR “Endogenous Nitrate Vasodilator” OR “Mononitrogen Monoxide”) AND (TITLE-ABS-KEY (malaria OR plasmodium OR "Plasmodium Infection" OR "Remittent Fever" OR "Marsh Fever" OR paludism))	1,196

Ovid 25 August 2023

No.	Key concept	Search terms	Results
1.	Nitric oxide AND Malaria	(“Nitric oxide” OR “Nitrogen Monoxide” OR “Endothelium-Derived Nitric Oxide” OR “Endogenous Nitrate Vasodilator” OR “Mononitrogen Monoxide”) AND (malaria OR plasmodium OR “Plasmodium Infection“ OR “Remittent Fever“ OR “Marsh Fever“ OR Paludism) {No Related Terms}	1429

ProQuest 25 August 2023

No.	Key concept	Search terms	Results
1.	Nitric oxide AND Malaria	(Ceruloplasmin OR “Ceruloplasmin Oxidase” OR Ferroxidase OR “alpha(2)-Ceruloplasmin” OR “Ceruloplasmin Ferroxidase” OR “Ferroxidase I”) AND (malaria OR plasmodium OR “Plasmodium Infection“ OR “Remittent Fever“ OR “Marsh Fever“ OR Paludism)	

Google Scholar

Google Scholar 29-7-2023

(malaria OR plasmodium) AND Nitric oxide
