# Supplementary material for: Association between Plasmodium Infection and Nitric Oxide Levels: A Systematic Review and Meta-Analysis
Source: Antioxidants (Basel). 2023 Oct 16;12(10):1868. doi: 10.3390/antiox12101868 (PMC10604424; doi:10.3390/antiox12101868)
Supplement: Supplementary file 1 [file antioxidants-12-01868-s001.zip › Table S4. Meta-regression results.pdf]

Association between *Plasmodium* infection and nitric oxide levels: A systematic review and meta-analysis

Kwuntida Uthaisar Kotepui[1†], Aongart Mahittikorn[2†], Polrat Wilairatana[3*], Frederick Ramirez Masangkay[4], Manas Kotepui[1]*

[1]Medical Technology, School of Allied Health Sciences, Walailak University, Thasala, Nakhon Si Thammarat 80160, Thailand

[2]Department of Protozoology, Faculty of Tropical Medicine, Mahidol University, Bangkok 10400, Thailand

[3]Department of Clinical Tropical Medicine, Faculty of Tropical Medicine, Mahidol University, Bangkok 10400, Thailand

[4]Department of Medical Technology, Faculty of Pharmacy, University of Santo Tomas, Manila 1000, Philippines

*Corresponding author

† These authors contributed equally to this work.

Kwuntida Uthaisar Kotepui: kwuntida.ut@wu.ac.th

Aongart Mahittikorn: aongart.mah@mahidol.ac.th

Frederick Ramirez Masangkay: frederick_masangkay2002@yahoo.com

Polrat Wilairatana: polrat.wil@mahidol.ac.th

Manas Kotepui manas.ko@wu.ac.th, Tel+ :.66954392469

Table S4. Meta-regression results

Meta-analysis of UA	Covariates	*P* value	tau2	I^2 (%)	R-squared (%)	Number of studies

**Malaria patients vs uninfected individuals**	Publication years	0.0004	1.145	97.31	0.00	12
	Study design	0.3785	1.14	97.55	0.00	12
	Continent	0.0038	1.509	98.06	0.00	12
	Age group	< 0.001	0.9001	97.02	11.66	12
	Plasmodium species	0.9013	1.769	97.94	0.00	12
	Diagnostic method for malaria	0.589	1.714	97.70	0.00	12
	Method for NO measurement	0.0193	1.378	97.81	0.00	12
	Types of blood samples	0.0258	1.444	97.77	0.00	12
